# Development and Validation of a Questionnaire to Assess Knowledge and Attitudes toward COVID-19 Preventive Measures in Romania

**DOI:** 10.3390/healthcare10101827

**Published:** 2022-09-21

**Authors:** Alina Delia Popa, Sabina Antonela Antoniu, Armand Iustinian Enache, Iolanda Valentina Popa, Raluca Alina Dragomir, Alexandru Burlacu

**Affiliations:** 1 Faculty of Medicine, University of Medicine and Pharmacy “Grigore T Popa”, 700115 Iasi, Romania; 2 Diabetes, Nutrition and Metabolic Disease Clinic, 700111 Iasi, Romania; 3 Clinic of Pulmonary Diseases, 700115 Iasi, Romania; 4 Institute of Cardiovascular Diseases “Prof. Dr. George I.M. Georgescu”, 700503 Iasi, Romania

**Keywords:** attitudes, COVID-19, hesitancy, prevention measures, vaccines, questionnaire, screening

## Abstract

The World Health Organization warns about the threat of the COVID-19 sixth wave. Our aim was to propose the first validated Romanian questionnaire to assess people’s level of education and attitudes towards general measures to protect against COVID-19 infection. Our study was conducted on a sample of 194 people. The first version of the questionnaire consisted of 40 items. Items that did not meet psychometric criteria were removed. Latent components/factors were identified through exploratory factorial analysis (EFA). The Cronbach’s alpha coefficient was used to assess internal fidelity. The EFA identified three factors. Factor 1 was named “Compliance with protective measures”, factor 2 was “Attitudes toward vaccination” and factor 3 was “Attitudes regarding potential COVID-19 therapies”. The final version of the questionnaire consists of 16 items. The test’s final score predicted the presence of vaccination with an accuracy of 0.773. The questionnaire score, the diagnosis of diabetes, the advice provided by healthcare workers and the medical profession proved to be significant predictors of vaccination. The implementation of our questionnaire within national programs could identify populational areas that need specific interventions to reach vaccination targets and prevent a full-blown sixth wave of the COVID-19 pandemic in Romania.

## 1. Introduction

The global COVID pandemic has affected more than half a billion people and has so far led to around 6,332,618 deaths, driving economic, political and social changes. The protective measures against SARS-CoV-2 virus infection are currently both general and recommended in all epidemics with respiratory-transmitted pathogens (social distancing, wearing protective masks), but also specific such as testing and vaccination. The role of these measures is to reduce the number of severe forms of disease and death but also to prevent the overcapacity of hospitalization and intensive care at the national level [1].

In a time of measures’ relaxation (the removal of the mandatory wearing of the mask in public places, the changes to the rules for testing and isolating new cases) after a period of decline in the incidence of COVID-19 cases throughout March, April and May 2022, our paper provides a warning signal based on latest World Health Organization (WHO) briefing (6 July 2022) that urges countries to readopt tried and tested public health measures and plug immunity gaps as infections new sub-lineages have risen 30% in the past two weeks (as of 6 July 2022) and increased in four out of six WHO regions [2]. This warning comes at a time when many countries have still not reached WHO’s vaccination targets (Bulgaria 29.9%, Slovakia 50.8%, Croatia 55.6%, Slovenia 57.1%, Poland 59.6%) [3] or gained access to antivirals. This is also the case for Romania, with a cumulative uptake of full vaccination in the total population of 42.3% as of 14 July 2022 [3]. Moreover, even for those vaccinated, vaccine efficiency declined with the appearance of new mutations in the structure of spike protein, thus amplifying the existent hesitancy in adherence to preventive measures [4]. Breakthrough infections in vaccinated populations with the recently emerged SARS-CoV-2 Omicron variant in the fifth wave of the pandemic exposed some of these limitations of COVID-19 vaccines and highlighted the need for other medical treatments such as drug therapy [5]. However, antiviral drugs prove to reduce the risk for severe outcomes and death in non-hospitalized patients or when given in the first 5 days after COVID diagnosis [6,7,8], but fewer data are on efficiency in hospitalized patients with severe disease [8]. These findings reinforce the need not to underestimate general preventive measures and the role of massive testing.

Worldwide, countries with high vaccination rates validated COVID-19 knowledge and behavior questionnaires (France [9], Spain [10], Italy [11], Hungary [12], Brazil [13], Korea [14], India [15], etc.). Timely insights from behavioral and attitudinal data proved to be crucial for the decision-making process in the COVID-19 international public health policies [16,17].

Currently, there is no validated Romanian questionnaire to assess people’s attitudes toward anti-COVID vaccination and general preventive measures. Such a tool is vital for improving people’s attitudes and compliance and reaching vaccination targets.

In this context, we considered the opportune warning from WHO, and we aligned ourselves to this call by proposing the first validated Romanian questionnaire to assess people’s level of education and attitudes towards general measures to protect against COVID-19 infection. Another objective of our study was to characterize the level of adherence and the main motivations behind acceptance or refusal to follow the imposed preventive measures. The ultimate goal was to use the proposed questionnaire within national programs to identify populational areas with low adherence and establish specific educational programs to increase the willingness to vaccinate against COVID-19 in an effort to prevent a full-blown sixth wave.

## 2. Materials and Methods

### 2.1. Questionnaire Development

We designed a survey tool, in the Romanian language, based on the guidelines provided by the World Health Organization Regional Office for Europe (WHO) [18] for the European member states that wish to conduct behavioral insights studies related to COVID-19.

The items of the questionnaire were proposed and reviewed by three physicians. The variables measured by this questionnaire addressed protective measures against COVID-19 infection: social distancing, masking, testing, vaccination, green certificate and following medical advice. As defined by the European Commission, the green certificate is “a proof that a person has been vaccinated against COVID-19, has received a negative test result or has recovered from COVID-19” [19].

The Likert scale was used to assess the answer to each question: 0—“Strongly Disagree”, 1—“Disagree”, 2—“Neither Agree nor Disagree”, 3—“Agree”, 4—“Strongly Agree”. The final score of the test represents the sum of all item scores. The lower the total score, the more people would tend to have a positive attitude toward vaccination and governmental measures.

### 2.2. Questionnaire Validation

The tested hypothesis was the following: patients with a better questionnaire score on the knowledge of preventive measures will have more frequent correct proangiogenic behavior.

After its development, the validation process followed several steps.

#### 2.2.1. Face Validity

Two experts evaluated the proposed items for their clarity, adequacy and significance in relation to appropriate measuring of knowledge and attitudes toward SARS-CoV-2 infection. Each item was evaluated by experts in relation to three standards:

- Relevance to the purpose of the questionnaire;
- Good matching with the recommendations provided to patients by national education programs;
- The relevance of the measures and behaviors we seek to assess.

Each of these three aspects, corresponding to an item, was assessed on a Likert scale from 1 to 5. Items considered by both experts to be unimportant were deleted.

The questionnaire was then tested for ease of use or ambiguity in a pilot test with ten nursing students. Items with an ambiguous definition of terms detected by the pilot test were removed or rephrased.

#### 2.2.2. Study Design and Participants

An observational study was then conducted on a convenience sample of 194 people. The criteria for inclusion was the completion of a Google form-based questionnaire distributed to the general population through social media (Facebook) between 7 March 2022 and 9 June 2022. All incomplete questionnaires were excluded. Respondents were not paid for their participation. The dissemination of the questionnaire drawn up on Google sheets was conducted through Facebook by the authors.

Approval from the Research Ethics Committee of the “Gr. T. Popa” University of Medicine and Pharmacy Iaşi (Nr. 155/23.02.2022) was obtained before contacting participants and collecting data. All participants provided signed informed consent. The informed consent was included as an introduction to the questionnaire. All data were anonymized to maintain participant confidentiality.

#### 2.2.3. Data Collection

Demographic data (age, gender, educational level, profession, medical history, vaccination history) and questionnaire answers revealing knowledge, attitudes and behaviors related to the COVID-19 outbreak were recorded for each responder.

#### 2.2.4. Psychometric Evaluation

We used psychometric criteria on the data obtained in the study to evaluate the following characteristics:

(a) Items to which at least 5% of people responded in the same way were retained [20,21];

(b) The discrimination index (DI) is a measure of how well an item can differentiate between good candidates and less able ones. For a specific item, DI, expressed as a percentage, was determined by calculating the difference between the number of people in the upper quartile and, respectively, in the lower quartile of the test’s total score, considering only responders who answered correctly to that item. Items with a coefficient of less than 20% do not have a sufficient capacity to discriminate between subjects and were therefore removed [21];

(c) The ability of each element to discriminate between people with different levels of knowledge was measured by correlating the score for each element with the overall test score (TSC—total score correlation). All items with significant correlations at a *p* < 0.001 were retained [21].

#### 2.2.5. Identifying Underlying Components

The next step was to identify latent components/factors through exploratory factorial analysis. Factorial analysis validates the construction of the questionnaire and reduces the number of variables to a number of components called factors that encompass a subset of the variables sharing a certain communality expressed through multicollinearity.

The Pearson correlation coefficient was determined for conducting the discriminative analysis. In order to evaluate construct validity, we first assessed the homogeneity of variance with the Levene test. Afterward, we determined whether there were significant differences in the mean scores using the ANOVA test. The construct validity was tested using exploratory analysis. The Kaiser–Meyer–Olkin measure of sampling adequacy (KMO) was used to signal in advance whether the sample size is large enough to extract factors reliably. The Bartlett’s Sfericity Test was used to assess the presence of multicollinearity, and the *p*-value of Bartlett’s test of sphericity was considered significant if it was lower than 0.05. The factor loading value of individual items is set to be greater than 0.25. We considered variables with a correlation coefficient greater than 0.4 as variables that contribute to the formation of a factor.

Principal component analysis (PCA) was used to reduce the dimensionality of the dataset in case of the presence of interrelated variables while retaining as much as possible of the variation.

Several indices were used to assess a good model fit for the construct, including the ratio of chi-square to degree of freedom (*χ*^2^/df) < 5.0, root mean square error of approximation (RMSEA) ≤ 0.08, comparative fit index (CFI) > 0.9, Tucker Lewis Index (TLI) > 0.9 and *p* > 0.05 for the chi-square test [22].

#### 2.2.6. Internal Fidelity

The Cronbach’s alpha coefficient was used to assess internal fidelity. The Guttman Split-Half coefficient showed the probability of test-retest reproducibility.

### 2.3. Statistical Analysis

The database containing the questionnaire responses was managed with Microsoft Office Excel 2007. Statistical processing was performed using the JASP 0.16.1 [15] and JAMOVI [14] software.

In order to test the normality of the distribution of the questionnaire scores, the Kolmogorov–Smirnov test was applied.

Sample size calculation was undertaken using the single proportion formula: *n* = Z_α/2_^2^·P·(1 − p)/d^2^ at 95% confidence interval, where Z_α/2_ = 1.96, P = prevalence of 50% and d = 5% of marginal error [23]. We also defined a reference group to estimate the sample size. Due to the lack of other available data, we used a population over 15 years old, with residency in the North East Region of Romania, reported on 1 July 2019 (*n* = 2,620,857) to define the size of the reference group [17]. Based on these calculations, the minimum sample size required was 370 (95% CI) or 165 (80% CI).

In order to emphasize the conformity and utility of our survey in screening for people’s attitudes toward vaccination, we performed both simple and forwarded binomial logistic regression to predict the vaccination status using the test’s score or other variables resulting from the factor analysis.

For ANOVA analyses, in the presence of two independent groups with α = 0.05 and a power of 95%, the sample size needed is estimated to be 210 participants. In the case of 190 participants, the power decreases to 93%. For logistical regression with 194 participants, OR = 1.3 and critical z 0.99, the power was estimated with the use of the G*Power 3.1.9.7 software at a value of 0.68.

## 3. Results

Our sample included 194 people, of which 137 (70.6%) were women, with a mean age of 41.5 years old and a standard deviation (SD) of 12.4. Age varied between 18 and 89 years old. Of all respondents, 58.2% inhabited urban areas. Regarding the geographical area, the participants came from the north-eastern part of Romania, and almost half of them were from Iasi county: Iasi 110, Bacau 14, Botosani 24, Galati 13, Neamt 12, Suceava 14, Vaslui 7. A significant proportion of the study sample declared to work in healthcare (27.53%) (Table 1).

The first version of the questionnaire reviewed by experts consisted of 40 items, to which the participants were instructed to respond according to their conviction (Table 2). The score for the items M2, M5, M6, M7, M8, T2, T4, V1, V2, V4, V5, V6, V7, V8, S1, S3, S4, S5, S6, S7, S8, CV2, CV3 and CV4 was reversed.

The mean score of the questionnaire was 42.8 (95% IC: 41–44.6; SD = 12.6). Significant differences in the score were observed between subgroups regarding the area of residence, the presence of diabetes, the awareness of the potential severity of SARS-CoV2 disease and the profession (Table 3). Vaccinated people had significantly lower scores.

According to the psychometric criteria, items SD1, M2, M3, M4, M6, V1, V2, V3, V4, V6, V10, S3, S4, S6 and S7 did not meet the recommendation that at least 5% of people should respond in the same way and were eliminated. Items M2, M3, M4, M6, V2, V3, V6, S3, S4, S6 and S7 were withdrawn due to the DI that was lower than 20%. Item V3 was removed due to the lack of ability to discriminate between people with different levels of knowledge.

**Figure 1:**
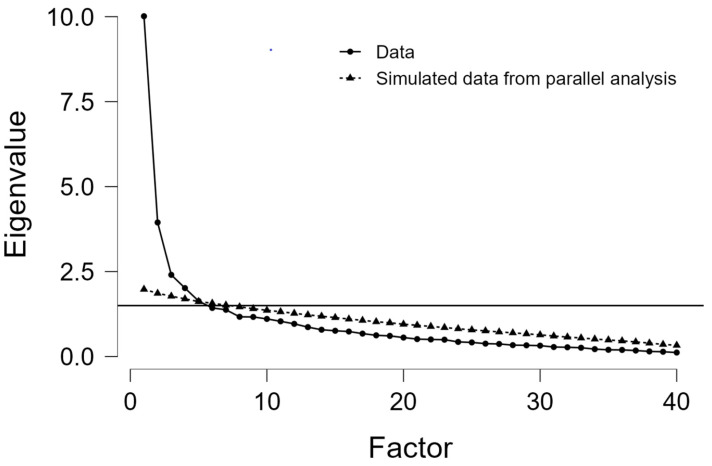
Scree plot of the eigenvalues of the factors identified in our questionnaire.

The exploratory factorial analysis for the identification of latent components identified three factors that together explained 52.7% of the total variation and were, therefore, chosen as the components of our questionnaire (Table 4, Figure 1). T The value of the Kaiser–Meyer–Olkin coefficient (0.791) and the result of Bartlett’s test (*χ*^2^ = 1571.865, *p* < 0.001) indicated the adequacy of the sample size chosen for our analysis. The names of the factors are given according to the variables that they encompass. Factor 1 was named “Compliance with protective measures”, factor 2 was “Attitudes toward vaccination” and factor 3 was “Attitudes regarding potential COVID-19 therapies”. For factor 1, there are high correlations between items exploring social distancing, testing and the protective role of mask-wearing. For factor 2, the items selected were addressing potential harmful side effects or inefficiency of the vaccines and attitudes toward restrictions associated with the green certificate. For factor 3, the questions investigated behaviors toward potential therapies for SARS-CoV-2 infection, uncertainty toward their scientific validation and distrust in the scientific plausibility of protective measures.

Principal component analysis (PCA) simplified the complexity of high-dimensional data while retaining trends and patterns. After performing PCA and excluding the variables with loadings lower than 0.5, the final model contains 16 items (Table 5 and Table 6) and confirms the factorial structure of the proposed questionnaire with the three dimensions identified by the exploratory factorial analysis.

The value of the alpha Cronbach coefficient shows a good internal fidelity of the questionnaire (0.853). The Guttman Split-Half coefficient shows the probability of test-retest reproducibility. By applying the Split-Half method to show the probability of test-retest reproducibility, a value of the Guttman Split-Half coefficient of 0.859 was obtained, which indicates that the fidelity of the scale is acceptable (Table 7).

**Figure 2:**
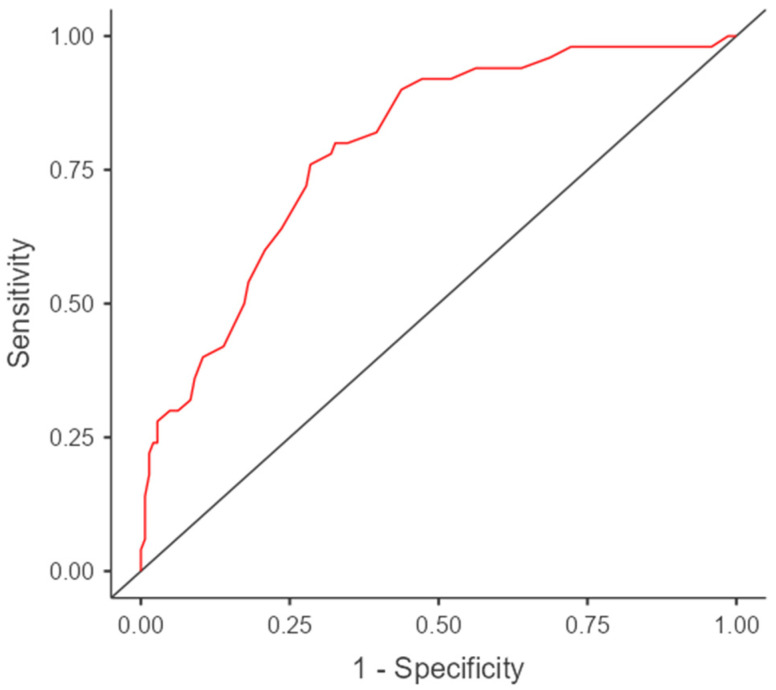
The performance of the test’s final score to predict the status of vaccination.

As another argument for the survey’s ability to screen people’s attitudes toward vaccination, we identified that the test’s final score predicts the presence of vaccination for a cut-off value of 50% with an accuracy of 0.773 and an AUC of 0.793 (Figure 2).

Table 8 illustrates the predictive power of various variables used in the factorial analysis to estimate the presence of vaccination. In total, the questionnaire score, the diagnosis of diabetes, the advice provided by healthcare workers and the medical profession proved to be significant predictors of vaccination.

## 4. Discussion

Our paper represents the first validation study of a Romanian questionnaire aimed at screening attitudes toward vaccination and preventive measures against SARS-CoV-2 infection. Our article contributes to the literature with a scientific method to validate a questionnaire in a non-English language. As such, our method can serve as a model to be used by researchers in other countries to develop and validate surveys in their language. Moreover, our article characterizes the level of adherence and the main motivations behind acceptance or refusal to follow the COVID-19 preventive measures of a Romanian population.

Even though we are now in a period of relaxation of the public health measures due to an apparent withdrawal of the epidemic in the first half of the year, recent publications warn about several countries entering the sixth wave of the epidemic [24,25,26,27]. According to the WHO’s latest briefing (6 July 2022), preventive measures and vaccination programs should again be implemented [2].

Several reviews dealing with the worldwide attitudes and hesitancy towards COVID-19 vaccination were published [28,29,30]. In countries with high levels of vaccination, knowledge and level of education proved to have positive associations with attitude and adherence to precautionary measures [23]. On the contrary, in Eastern Europe, a lower overall proportion of vaccine acceptance has been reported [30]. Increasing the vaccination rate is crucial in combating the COVID-19 pandemic, but it requires the prior identification of the underlying causes/specific determinants of hesitancy towards vaccines specific to this region.

Similar to other middle-income countries, Romania faces several barriers and difficulties in re-establishing control measures against the COVID-19 epidemic. The means of controlling the SARS-CoV-2 virus infection spreading were accompanied by economic costs such as unemployment, corporate bankruptcies and a disproportionate impact on less-skilled and less-educated workers. Social costs included increased domestic violence and damaged educational systems [31]. Furthermore, the media attention and uncertainty about how to protect against infection generate contradictory attitudes and behaviors among the population, starting from accepting protective measures and vaccination to denying their effects and even the disease. Along with the conspiracy theories, the imposition of compulsory vaccination has increased social tensions in many countries, with vaccination being associated with the threat to individual human freedoms, such as the right to free choice of health or the right to work. This perception of the threat to freedom has provided an opportunity for various political factions to condemn vaccination, to question the effectiveness of current vaccines amid the tension generated by general protection measures seen as restrictive in order to gain electoral capital. These perspectives emphasize that while COVID-19 vaccines are becoming increasingly available, they are still met with reluctance, and thus, safety measures (e.g., face masks, personal hygiene and social distancing) are still of key importance in protecting personal and public health against COVID-19 [32].

In order to meet the WHO’s recommendations and overcome the particular Romanian patterns of public health measures adherence and acceptance, we developed and validated a questionnaire that addresses knowledge, attitudes and practices focusing on preventive measures against COVID-19. It is divided into six categories related to social distancing, mask-wearing, testing, vaccination, use of supplements and the benefit of a green certificate.

The analysis of the completed questionnaire database offers a global picture of the adherence motivations to general preventive measures against COVID-19 in Romania. By knowing the actual reasons behind not reaching the country’s vaccination target, specific measures can be taken to increase people’s confidence and compliance. For instance, cardiovascular patients within our 194-sized sample did not have higher vaccination rates, although cardiovascular diseases are known risk factors for developing severe forms of COVID-19 [33,34,35,36]. However, the presence of diabetes was significantly associated with a better score in our questionnaire and with a higher proportion of vaccinated people. Thus, specific measures directed toward cardiovascular patients to raise awareness through educational programs could be effective in levering up vaccination rates. Other main determinants of vaccine acceptance and preventive measures compliance, as identified by our study, were related to vaccines’ safety and efficiency, the people’s trust in the government and medical system, and health literacy. These results show us that there is room for progress in COVID-19 health literacy through intensified education. Gaining people’s trust in the government and healthcare system through communication and education should also be a priority. Our results also suggest a need for targeted community awareness interventions for the most vulnerable populations, those with no school education, the elderly and people living in rural areas.

Moreover, we conducted a multivariate analysis that identified several predictors of vaccination adherence: questionnaire’s final score, medical profession, medical advice versus mass media consultation, the presence of diabetes mellitus and the fear of the disease.

In addition to proposing a Romanian survey regarding COVID-19 attitudes, we also proceeded to the scientific validation of the questionnaire in order to obtain pertinent information in a reliable and valid way [21] and ensure that the questionnaire “*measures what is intended to be measured*” [37]. The application of improper measurement tools that are not validated can lead to inaccurate and misleading findings, resulting in a poor plan for interventions and, therefore, too unreliable efficacy.

Validity is assessed through two categories of tests, which evaluate theoretical construct and empirical construct. The theoretical construct is tested through face validity. The purpose of face validity is to ascertain that the items of the questionnaire fully represent the domain that is intended to be judged [37]. We used literature reviews, critical incidents, direct observations and expert judgment approaches to construct a questionnaire of 40 items with acceptable face validity. The literature review did not identify, to date, any similar validated questionnaire in the Romanian language [38]. Face validity is an important aspect related to the empirical construct of the questionnaire [20,21]. The next validation step was represented by the psychometric evaluation, which prompted the elimination of 15 questionnaire items due to not meeting psychometric criteria. The remaining 25 items were included in the exploratory factor analysis. The factor analysis revealed a three factors structure with 20 items. The resulting model explained 52.7% of the variance in our study. Furthermore, PCA was applied, resulting in a final model of 16 items.

In order to strengthen the evidence that the questionnaire “*measures what is intended to be measured*”, we proved that the test is able to discriminate between vaccinated and unvaccinated people.

Our results are similar to other resembling COVID-19 questionnaire validations in other languages. A Korean study was based on similar sample size (229) and obtained an alpha Cronbach coefficient of 0.75 for validating a survey that assesses COVID-19 knowledge, attitudes and practices among nursing students [39]. Likewise, the validation of an Indian questionnaire to assess knowledge, attitude, practices and concerns regarding COVID-19 vaccination among the general population was based on 201 participants and resulted in an alpha Cronbach coefficient of 0.86. With the exception of a french questionnaire aimed to assess COVID-19 knowledge and behaviors that was validated on a big cohort of 8045 participants [9], our study fits the current international scientific landscape for the population sample as well as validity results.

### Limitations

Firstly, the number of survey respondents was small. This low interest may be explained by the fact that the incidence of COVID-19 cases declined during the spring of 2022, preventive measures were relaxed, and the mass media and people’s attention was focused on the war in Ukraine, with which Romania shares a significant long border. However, this number is still appropriate for factor analysis and testing of the questionnaire. Secondly, our sample might not be representative of the general population in Romania as the proportion of vaccinated people was 74,8%, much higher than that reported by the authorities of 42.3% [3] (as of 14 July 2022). Moreover, a significant proportion were healthcare workers, thus explaining the high rate of vaccination. The percentage of women (70.6%) in our sample is also higher compared to the general population. In our future validation studies, we aim to overcome these limitations by including a larger population with similar variables’ distributions as the general population.

## 5. Conclusions

In the context of the re-emergence of new variants of SARS-CoV-2 spreading globally, with steady recommendations from the WHO to re-establish some general preventive measures and reinforce vaccination programs, this study aimed to achieve the following: (1) propose the first validated Romanian questionnaire aimed at screening attitudes toward COVID-19 measures; (2) identify the main motivations to accept or reject anti-COVID-19 measures in a Romanian population sample; and (3) pave the way to the integration of our questionnaire as part of the national programs to take the pulse of people’s attitudes, raise awareness and identify populational areas that need specific interventions, in order to reach vaccination targets and prevent a full-blown sixth wave of the COVID-19 pandemic in Romania.

## Figures and Tables

**Table 1:** Characteristics of the study sample.

Socio-Demographical Characteristic		Count	% of Total		N	% of Total
Gender	F	137	70.6	M	57	29.4
Area of residence	Rural	81	41.8	Urban	113	58.2
Profession	Healthcare	59	27.353	Other	135	72.7
Formal education	Secondary/high-school	107	54.87	Bachelor or more	88	45.12
Cardiovascular disease	Yes	42	21.6	No	152	78.4
Pulmonary diseases	Yes	13	6.7	No	181	93.3
Diabetes mellitus	Yes	61	31.4	No	133	68.6
Smoking	Yes	61	31.4	No	133	68.6
Allergy	Yes	35	18	No	159	82
Vaccinated	Yes	144	74.2	No	50	25.8
Type of vaccine	BNT162b2	119	82.6			
	mRNA-1273	3	2.08			
	AZD1222 (ChAdOx1)	9	6.25			
	NJ-78436735 (Ad26.COV2.S)	13	9.02			
History of COVID-19	Yes	98	50.5	No	96	49.5
Hospitalised	Yes	16	16.32	No	82	83.67
Supplements	Yes	118	45.36	No	76	39.17
	Vitamin D	12	10.16			
	Zinc	3	2.54			
	Vitamin C	17	14.40			
	Multivitamins	86	72.88			
COVID-19 treatment	Umifenovir	5	5.1	No	32	32.65
	Favipiravir	2	2			
	Ivermectin	1	1.02			
	Recommended by the doctor	58	59.18			
Source of advice	Facebook	10	5.2			
	Family	11	5.7			
	Internet	74	38.1			
	Family doctor	27	13.9			
	TV	34	17.5			
	Friends	4	2.1			
	Other physician	34	17.5			
COVID-19 can put me at risk?	Yes	104	53.6	No	90	46.4
When I think about the possibility of getting COVID-19, I feel worried.	Yes	78	40.2	No	116	59.8

**Table 2:** First version of the questionnaire.

			Answer Variants: Number of Respondents (Percentage)						
	Code	Item	1	2	3	4	5	TSC	DI
1	SD1	Adherence to social distancing can prevent SARS-CoV-2 infection	94 (48.5)	40 (20.6)	41 (21.1)	6 (3.1)	13 (6.7)	0.608	20.61
2	SD2	The recommended distance to avoid the spread of the virus is 1.5 m	76 (39,2)	53 (27,3)	38 (19.6)	13 (6.7)	14 (7.2)	0.454	27.31
3	SD3	I believe that I have followed this recommendation most of the time	58 (29.9)	58 (29.9)	38 (21.6)	13 (9.3)	14 (7.2)	0.458	29.89
4	SD4	I avoided crowded areas	64 (33)	45 (23.2)	44 (22.7)	23 (11.9)	16 (9.3)	0.399	23.19
5	M1	Wearing a mask prevents SARS-CoV-2 infection	83 (42.8)	43 (22.2)	38 (19.6)	11 (5.7)	19 (9.8)	0.586	22.16
6	M2	The mask is correctly worn by covering only the mouth	34 (17.5)	7 (3.6)	8 (4.1)	14 (7.2)	131 (67.5)	0.461	11.34
7	M3	The mask is correctly worn by covering both the nose and the mouth	156 (80.4)	33 (17)	22 (11.3)	8 (4.1)	30 (15.5)	0.536	0
8	M4	I wear the mask correctly in public/crowd most of the time	101 (52.1)	33 (17)	22 (11.3)	8 (4.1)	30 15.5)	0.522	11.34
9	M5	I can’t stand the mask because I feel suffocated/can’t speak or hear well	33 (17)	19 (9.8)	23 (12.9)	25 (12.9)	94 (48.5)	0.488	24.74
10	M6	I can’t wear the mask because of comorbidities	29 (14.9)	8 (4.1)	10 (5.2)	9 (4.6)	138 (71.1)	0.518	9.79
11	M7	The mask prevents me from recognizing the people around me	41 (21.1)	28 (14.4)	36 (18.6)	25 (12.9)	64 (23)	0.551	31.4
12	M8	Children should not wear a mask	41 (21.1)	23 (11.9)	40 (20.8)	26 (13.4)	63 (32.3)	0.544	34.02
13	T1	I agree with PCR testing	91 (46.9)	41 (21.1)	30 (15,5)	10 (5.2)	22 (11.3)	0.544	21.1
14	T2	Nasal tests are invasive	36 (18.6)	24 (12.4)	47 (24.2)	25 (12.9)	62 (32)	0.302	37.11
15	T3	The RT-PCR test is based on the detection of viral RNA	76 (39.2)	38 (19.6)	50 (25.8)	11 (5.7)	19 (9.8)	0.405	45.3
16	T4	Many RT-PCR tests are false	35 (18)	19 (9.8)	38 (19.6)	44 (22.7)	58 (29.9)	0.557	42.26
17	T5	I agree with rapid tests	73 (37.6)	42 (21.6)	44 (22.7)	13 (6.7)	22 (11.3)	0.498	21.64
18	T6	Rapid tests can give erroneous results	73 (37.6)	49 (25.3)	49 (25.3)	14 (7.2)	9 (4.6)	0.253	25.25
19	V1	I didn’t get vaccinated because it affects my freedom	32 (16.5)	6 (3.1)	16 (8.2)	13 (6.7)	127 (65.5)	0.600	14.94
20	V2	I didn’t get vaccinated because it changes my DNA, and it’s an experiment on the population	32 (16.5)	7 (3.6)	18 (9.3)	12 (6.2)	125 (64.4)	0.614	15.46
21	V3	I got vaccinated for my protection and to protect those around me	109 (56.2)	18 (9.3)	26 (13.4)	6 (3.1)	35 (19)	0.092	9.27
22	V4	I did not get vaccinated because the vaccine is not tested enough, and I am waiting to see what happens to others	43 (22.2)	7 (3.5)	20 (10.3)	16 (8.2)	108 (55.7)	0.399	13.9
23	V5	I got vaccinated so as not to have problems at work or to be able to travel abroad	43 (22.2)	24 (12.)	41 (21.1)	12 (6.2)	74 (38.8)	0.488	27.31
24	V6	I did not get vaccinated because I have chronic diseases or allergies.	18 (9.3)	5 (2.6)	20 (10.3)	9 (4.6)	142 (73.2	0.481	4.63
25	V7	Vaccinated people transmit the disease more or similar to unvaccinated people	41 (21.1)	14 (7.2)	58 (29.9)	26 (13.4)	54 (27.8)	0.471	58,13
26	V8	The vaccine can cause heart disease (myocarditis/endocarditis)/thrombosis)	36 (18.6)	27 (13.9)	53 (27.3)	29 (14.9)	49 (25.3)	0.523	53.14
27	V9	The vaccine does not provide protection against infection	49 (25.3)	30 (15.5)	41 (21.1)	30 (15.5)	44 (22.7)	0.296	36.59
28	V10	The vaccine prevents severe or fatal COVID-19	135 (69.8)	54 (27.8)	0	3 (1.5)	2 (1)	0.523	81
29	S1	Foods rich in vitamin C surely prevent COVID-19	20 (10.3)	20 (10.3)	64 (33)	46 (23.7)	44 (22.7)	0.296	32.98
30	S2	Vitamin D has an important role in disease prevention	27 (13.9)	34 (17.5)	76 (39.3)	28 (14.4)	29 (14.9)	0.172	39.17
31	S3	Quercetin is a drug that cures COVID-19	13 (6.7)	11 (5.7)	97 (50)	24 (12.4)	49 (25.3)	0.454	12.37
32	S4	Zinc cures COVID-19	18 (9.3)	10 (5.2)	68 (35.1)	30 (15.5)	68 (35.1)	0.528	15.46
33	S5	The state does not provide drugs that cure COVID-19	33 (17)	27 (13.9)	62 (32)	38 (19.6)	34 (17.5)	0.481	31.95
34	S6	Arbidol is effective in curing COVID-19	28 (14.4)	21(10.8)	98 (50.5)	22 (11.3)	25 (12.9)	0.397	10.82
35	S7	Although Arbidol is very good, it is not brought to hospitals	24 (12.4)	22 (11.3)	106 (54.6)	19 (9.8)	23 (11.9)	0.505	0
36	S8	Ivermectin is effective in curing COVID-19	21 (10.8)	13 (6.7)	108 (55.7)	17 (8.8)	35 (18)	0.382	22.68
37	CV1	The green certificate is a measure to control the spread of the infection	56 (28.9)	34 (17.5)	35 (18)	17 (8.8)	52 (26.8)	0.548	44.32
38	CV2	I don’t think using the green certificate prevents the infection from spreading	62 (32)	29 (14.9)	30 (15.5)	27 (13.9)	44 (22.7)	0.580	59.13
39	CV3	The green certificate is a measure against the freedom of individuals	51 (26.3)	21 (10.8)	35 (18)	19 (9.8)	68 (35.1)	0.627	35.1
40	CV4	The green certificate should not be used at this time	66 (34)	19 (9.8)	35(18)	22 (11.3)	52 (26.4)	0.614	35.09

TSC—total score correlation; DI—discrimination index.

**Table 3:** Differences in score between subgroups.

Socio-Demografic Breakdown of Study Sample		N	Mean	SD	Cohen’s d	*p*
Gender	Feminine	137	42.57	12.72	0.06	0.682
	Masculine	57	43.42	13.32		
Area of residence	Rural	81	45.00	12.65	0.29	0.046
	Urban	113	41.26	12.86		
Cardiovascular diseases	Yes	42	43.59	11.04	0.07	0.628
	No	152	42.61	13.36		
Diabetes	Yes	39	46.30	11.89	0.34	0.049
	No	105	41.94	13.00		
COVID infection is a threat for me	Yes	104	39.59	11.25	0.55	<0.001
	No	90	46.55	13.66		
Vaccinate	Yes	144	39.35	11.26	1.15	<0.001
	No	50	52.82	12.06		
History of COVID infection	Yes	98	43.02	11.97	0.02	0.832
	No	96	42.65	13.79		
Medical Profession	Yes	135	38.65	11.23	1.31	<0.001
	No	59	53.37	11.26		
Source of advice	Family and friends	15	38.60	10.02		0.08
	Mass media	117	44.36	12.49		
	Medical professionals	62	40.98	13.87		

**Table 4:** The 3 identified components/factors of the questionnaire along with the variables that they encompass.

Item	Factor 1	Factor 2	Factor 3
SD4	0.777		
SD3	0.744		
SD2	0.650		
T1	0.630		
M1	0.612		
T5	0.538		
V8		0.800	
V7		0.659	
CV2		0.601	
CV3		0.566	
V9		−0.535	
CV4		0.504	
CV1		0.453	
S1			0.690
S2			−0.619
M8			0.493
S5			0.439
S8			0.417
M7			0.416

T2, T3, T4, T6 and V5 have factor loadings lower than 0.4.

**Table 5:** Goodness of fit indices for the final model.

Factors	No of Items	Model Fit for the Construct		Goodness of Fit Indices				
		*χ2* (df)	*p* Value	df	CFI	TLI	RMSEA	SRMR
Model 1	18	397.0 (164)	<0.001	164	0.811	0.781	0.086	0.086
Model 2	15	176.76 (112)	<0.001	112	0.938	0.925	0.055	0.056

CFI—comparative fit index; TLI—Tucker Lewis Index; RMSEA—root mean square error of approximation; SRMR—standardized root mean squared error.

**Table 6:** Final model’s factor loadings.

Factor	Indicator	Symbol	Estimate	Std. Error	z-Value	*p*	95% Confidence Interval		Std. Est. (All)
							Lower	Upper	
1	SD3	λ11	0.725	0.091	7.974	<0.001	0.547	0.903	0.577
	SD4	λ12	0.726	0.095	7.632	<0.001	0.539	0.912	0.557
	T1	λ13	0.953	0.093	10.206	<0.001	0.770	1.136	0.704
	T5	λ14	0.865	0.094	9.179	<0.001	0.680	1.049	0.647
	M1	λ15	0.966	0.089	10.908	<0.001	0.793	1.140	0.741
	SD2	λ16	0.724	0.087	8.275	<0.001	0.552	0.895	0.594
2	V8	λ21	0.612	0.106	5.776	<0.001	0.404	0.820	0.434
	V9	λ22	−0.704	0.111	−6.370	<0.001	−0.921	−0.487	−0.472
	CV1	λ23	1.013	0.110	9.220	<0.001	0.798	1.229	0.644
	CV2	λ24	1.134	0.108	10.503	<0.001	0.922	1.345	0.713
	CV3	λ25	1.112	0.112	9.941	<0.001	0.893	1.331	0.684
	CV4	λ26	1.188	0.109	10.931	<0.001	0.975	1.401	0.736
	V7	λ27	0.637	0.111	5.761	<0.001	0.420	0.854	0.432
3	M7	λ31	1.015	0.122	8.328	<0.001	0.776	1.254	0.666
	M8	λ32	1.020	0.122	8.367	<0.001	0.781	1.259	0.669
	S5	λ33	0.625	0.106	5.894	<0.001	0.417	0.833	0.478

**Table 7:** Frequentist scale reliability statistics.

Estimate	Cronbach’s α	Guttman’s λ2	Guttman’s λ6	Mean	SD
Point estimate	0.853	0.859	0.885	42.825	12.877
95% CI lower bound	0.820	0.822	0.865	41.013	11.710
95% CI upper bound	0.881	0.887	0.915	44.637	14.303

**Table 8:** Role of variables in predicting vaccination status.

Predictor	Estimate	SE	Z		*p*	Odds Ratio	95% Confidence Interval	
							Lower	Upper
Intercept	−6.6360	1.2136	−5.468		<0.001	0.00131	<0.001	0.0142
Sum Score	0.0807	0.0213	3.793		<0.001	1.08403	1.040	1.1302
Area of residence								
Urban–Rural	−0.1273	0.4311	−0.295		0.768	0.88050	0.378	2.0498
Diagnosis of diabetes								
Yes–No	1.2328	0.4924		2.503	0.012	3.43092	1.307	9.0071
COVID-19 can endanger me?								
Yes–No	0.2908	0.4651	0.625		0.532	1.33749	0.538	3.3280
Where did you find out the most about COVID infection?								
1–2 (family–mass-media)	0.1292	0.8763	0.147		0.883	1.13790	0.204	6.3393
3–2 (medical workers–media)	1.7884	0.5239	3.414		<0.001	5.98012	2.142	16.6975
Profession (medical–nonmedical)								
2–1	1.9658	0.4850	4.053		<0.001	7.14074	2.760	18.4760

SE = standard error of the coefficient estimates.

## Data Availability

Data used in the study will be available from the corresponding authors upon request.
